# Two new competing pathways establish the threshold for cyclin-B–Cdk1 activation at the meiotic G2/M transition

**DOI:** 10.1242/jcs.182170

**Published:** 2016-08-15

**Authors:** Daisaku Hiraoka, Ryota Aono, Shin-ichiro Hanada, Eiichi Okumura, Takeo Kishimoto

**Affiliations:** 1 Science and Education Center, Ochanomizu University, Tokyo 112-8610, Japan; 2 Laboratory of Cell and Developmental Biology, Graduate School of Bioscience, Tokyo Institute of Technology, Yokohama 226-8501, Japan

**Keywords:** Gγ, G_β_, Oocyte, PI3K, Akt, Threshold, Cyclin B, Cdk1, Meiotic G2/M transition

## Abstract

Extracellular ligands control biological phenomena. Cells distinguish physiological stimuli from weak noise stimuli by establishing a ligand-concentration threshold. Hormonal control of the meiotic G2/M transition in oocytes is essential for reproduction. However, the mechanism for threshold establishment is unclear. In starfish oocytes, maturation-inducing hormones activate the PI3K–Akt pathway through the G_βγ_ complex of heterotrimeric G-proteins. Akt directly phosphorylates both Cdc25 phosphatase and Myt1 kinase, resulting in activation of cyclin-B–Cdk1, which then induces meiotic G2/M transition. Here, we show that cyclin-B–Cdk1 is partially activated after subthreshold hormonal stimuli, but this triggers negative feedback, resulting in dephosphorylation of Akt sites on Cdc25 and Myt1, thereby canceling the signal. We also identified phosphatase activity towards Akt substrates that exists independent of stimuli. In contrast to these negative regulatory activities, an atypical G_βγ_-dependent pathway enhances PI3K–Akt-dependent phosphorylation. Based on these findings, we propose a model for threshold establishment in which hormonal dose-dependent competition between these new pathways establishes a threshold; the atypical G_βγ_-pathway becomes predominant over Cdk-dependent negative feedback when the stimulus exceeds this threshold. Our findings provide a regulatory connection between cell cycle and signal transduction machineries.

## Abstract (precis)

**Summary:** Ligand–dose thresholds control ligand-dependent responses. To establish the hormonal threshold for driving meiosis, a stimulus-dependent positive regulatory pathway competes against negative feedback from cell cycle machinery.

## INTRODUCTION

Cells must respond to extracellular stimuli, such as hormones and environmental stresses, in order to adapt to changing environments. By establishing a stimulus threshold, cells are able to discriminate physiologically relevant stimuli from noise stimuli. Supra-threshold but not subthreshold doses of stimuli elicit cellular responses. Such stimulus-intensity-dependent responses can control progression of the cell cycle. An important example is hormonal control of meiosis in oocytes. In most animals, immature oocytes, which have a large nucleus, called the germinal vesicle, arrest at prophase of meiosis I (meiotic prophase I) (Masui, 1985), which is equivalent to the G2/M-phase border in somatic cells. Extracellular stimuli through maturation-inducing hormones then induce transition from meiotic prophase I to metaphase I through activation of cyclin-B–Cdk1 (equivalent to the G2/M transition in somatic cells) (Kishimoto, 2003), hallmarked by germinal vesicle breakdown (GVBD). Failure to tightly control this transition could result in serious defects in reproduction. Previous studies in frog and starfish have focused on signaling induced by supra-threshold doses of hormones (Haccard and Jessus, 2006; Kishimoto, 2003). However, the mechanism by which subthreshold stimuli are prevented from eliciting transition remains unclear. Thus, the nature of threshold-setting for hormone-dependent regulation of the meiotic G2/M transition is not fully understood. Here, we studied the molecular mechanism of threshold-setting using starfish oocytes, a model that has been widely used for studying G2/M transition (Kishimoto, 2015).

In starfish oocytes, the maturation-inducing hormone 1-methyladenine (1-MeAde) induces the meiotic G2/M transition (Kanatani et al., 1969; Kishimoto, 2011), with no requirement for new protein synthesis (Dorée, 1982). Based on previous studies using supra-threshold doses of 1-MeAde, the following model of the signaling has been established. In immature oocytes, the kinase Myt1 (UniProt ID Q99640 for the human form) inhibits cyclin-B–Cdk1 by phosphorylating residues Thr14 and Tyr15 on Cdk1 (Okumura et al., 2002). 1-MeAde promotes dissociation of the G_βγ_ complex (comprising the G_β_ and G_γ_ subunits; hereafter referred to as G_βγ_) of heterotrimeric G-proteins from G_i_α (Chiba et al., 1993; Jaffe et al., 1993; Shilling et al., 1989) through a putative 1-MeAde receptor located on the oocyte plasma membrane (Kanatani and Hiramoto, 1970). The G_βγ_ complex then binds to and activates PI3K, which produces phosphatidylinositol 3,4,5-triphosphate (PIP_3_) at the plasma membrane (Sadler and Ruderman, 1998; Vanhaesebroeck et al., 2010). In a PIP_3_-dependent manner, phosphoinositide-dependent kinase 1 (PDK1) and target of rapamycin complex 2 (TORC2) phosphorylate Akt on its activation loop and C-terminal hydrophobic motif, respectively, thereby fully activating Akt (Hiraoka et al., 2004, 2011). Akt directly inhibits Myt1 by phosphorylating residue Ser75 (Okumura et al., 2002). Cdc25, the phosphatase for Myt1 sites on Cdk1, is thought to be phosphorylated by Akt (Okumura et al., 2002), although the role of this phosphorylation remains unclear. Consequently, Cdc25 activity predominates over that of Myt1, resulting in cyclin-B–Cdk1 activation through dephosphorylation of Cdk1 (Okumura et al., 1996, 2002). Cyclin-B–Cdk1-dependent positive feedback further activates Cdc25 and inactivates Myt1 through phosphorylation of residues other than Akt sites (Hara et al., 2012; Kishimoto, 2015; Okumura et al., 2014). Finally, fully activated cyclin-B–Cdk1 causes an irreversible meiotic G2/M-phase transition.

The PI3K–Akt pathway is also essential in various signal transduction events induced by ligands such as insulin, growth factors and neurotransmitters in somatic cells (Alessi et al., 1997a,b; Hoeffer and Klann, 2010; Manning and Cantley, 2007; Sarbassov et al., 2005). Akt phosphorylates diverse substrates and thereby regulates cellular functions, including cell survival, glucose metabolism and cell cycle control (Manning and Cantley, 2007). In these cases, pathways downstream of Akt further affect PI3K activity, forming feedback loops. For example, Akt-dependent activation of mammalian target of rapamycin complex 1 (mTORC1) leads to suppression of transcription of PDGF receptor (Zhang et al., 2007). In insulin signaling, the adaptor protein insulin receptor substrate 1 (IRS1) mediates PI3K activation (Esposito et al., 2001; Sun et al., 1991). Akt indirectly activates p70S6K, which then phosphorylates IRS1, thereby suppressing PI3K activation (Harrington et al., 2004). To date, no such regulatory signaling networks have been reported in 1-MeAde signaling in starfish oocytes.

In this study, our initial analysis focused on the signaling dynamics induced by subthreshold levels of 1-MeAde. We found that such stimuli initiate activation of cyclin-B–Cdk1 but that the cyclin-B–Cdk1-dependent negative-feedback pathway causes rapid dephosphorylation of Akt substrates, including Cdc25 and Myt1, thereby canceling signaling by subthreshold stimuli. This implied the existence of an overriding mechanism that counteracts this negative feedback and is thereby able to fully activate cyclin-B–Cdk1 in response to supra-threshold levels of 1-MeAde. Consistently, we identified a novel ‘atypical G_βγ_-pathway’ in which G_βγ_ enhances PI3K–Akt-dependent phosphorylation of Cdc25 and Myt1. Furthermore, we found that, in addition to acting as an overriding mechanism, this atypical pathway is also required for the PI3K–Akt pathway to induce phosphorylation of Akt substrates even in the absence of the negative feedback. Based on these findings, we propose a model for full activation of cyclin-B–Cdk1 at the meiotic G2/M transition in which competition between newly identified mutually opposing pathways establishes the 1-MeAde-dose threshold.

## RESULTS

### Ser188 on Cdc25 is phosphorylated by Akt

To monitor signaling dynamics, we performed immunoblotting with phosphorylation (phospho)-specific antibodies against residue Ser477 (the TORC2 phosphorylation site) on Akt (Hiraoka et al., 2011) and residue Tyr15 on Cdk1 to monitor the activity of cyclin-B–Cdk1. In addition, to monitor Akt substrate phosphorylation, we generated phospho-specific antibodies against Myt1 and Cdc25. The anti-phospho-Myt1 (residue Ser75) antibody detected phosphorylation of an exogenously introduced Myt1-derived peptide containing Ser75 (Myt1-S75-peptide) in starfish oocytes (Fig. S1A) but was not suitable for detecting endogenous Myt1 owing to high background and reactivity with non-specific bands (Fig. S1B). With respect to Cdc25, Akt phosphorylated and activated recombinant starfish Cdc25 *in vitro* (Fig. S1C). Cdc25 contains five Akt consensus sites (RXRXXS/T, where X is any amino acid), and we successfully generated an antibody able to detect endogenous Cdc25 phosphorylation on residue Ser188 following 1-MeAde stimulation (Fig. S1B). Inhibition of Akt activation by an anti-TOR neutralizing antibody (recognizing starfish TOR, which is a catalytic subunit of TOR complexes) (Hiraoka et al., 2011) disrupted this phosphorylation, suggesting that Cdc25 is phosphorylated at Ser188 by Akt in starfish oocytes (Fig. S1D).

### Subthreshold levels of 1-MeAde trigger cyclin-B–Cdk1 activation, but a subsequent dephosphorylation of Akt substrates cancels the signaling

**Fig. 1.**
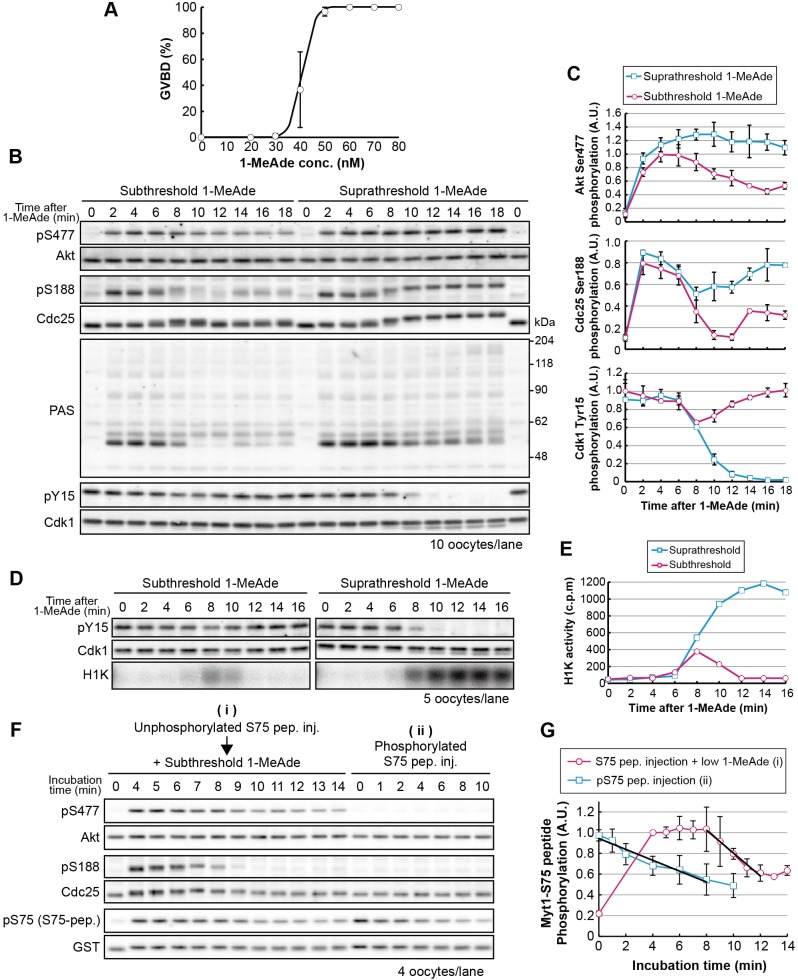
**Signaling by** **subthreshold levels of** **1-MeAde is canceled through the characteristic dephosphorylation of Akt substrates, including Cdc25.** (A) 1-MeAde dose-response curve in starfish oocytes. Thirty immature oocytes were treated with various concentrations of 1-MeAde. After 90 min, the proportion of oocytes that had undergone GVBD, a marker of the meiotic G2/M-phase transition that occurs at approximately 18 min after 1-MeAde addition, were counted. Data represent mean values±s.d. from three independent experiments. (B) Immature oocytes were treated with sub- (30 nM) or supra-threshold (500 nM) concentrations of 1-MeAde, collected at the indicated times and subjected to immunoblot with antibodies against phospho-Akt (at Ser477; pS477), Akt (Akt), phospho-Cdc25 (pS188), Cdc25 (Cdc25), pan phospho-Akt substrates (PAS), phospho-Cdk1 (at Tyr15; pY15) or the PSTAIR epitope (Cdk1). (C) The signal intensity of each band in B was quantified and normalized against a standard sample [an aliquot of a batch sample of 1-MeAde-treated (500 nM, 3 min) oocytes was loaded on an extra lane (not shown) as a standard in every independent experiment]. Phosphorylation levels (pS477, pS188 and pY15) were further normalized against total amounts of each protein (Akt, Cdc25 and Cdk1, respectively). Data represent mean values±s.d. from three independent experiments. (D,E) Immature oocytes were treated with sub- (40 nM) or supra-threshold (500 nM) concentrations of 1-MeAde, followed by analysis with the H1 kinase assay [autoradiography (D; H1K) and liquid scintillation counting (E)] and immunoblot. The data shows a representative of two independent experiments. (F) Immature oocytes were injected with the Myt1-S75 peptide (S75 pep.) followed by treatment with subthreshold (7 nM) levels of 1-MeAde (i), or the Myt1-S75 peptide was phosphorylated by human Akt1 *in vitro* and then injected into immature oocytes (ii). After incubation, the oocytes were collected at the indicated times and subjected to immunoblot analysis to detect phosphorylation and total protein levels. (G) Phosphorylation levels of the Myt1-S75-peptide were quantified from the immunoblot images shown in F and normalized against the total amount of each protein. The phosphorylation levels, relative to those at 4 min in ‘i’, are indicated as the mean value±s.d. from three independent experiments. Black lines indicate the linear approximation to show the dephosphorylation rate. inj., injection.

To elucidate the threshold-setting mechanism, we first compared 1-MeAde signaling dynamics between supra- and subthreshold stimuli. Fig. 1A shows a typical dose–response curve. The highest concentration at which GVBD failed to occur in any oocytes was used as the subthreshold 1-MeAde dose (Fig. 1A; 30 nM). A supra-threshold dose of 1-MeAde (500 nM) induced phosphorylation of Akt and Cdc25 within 2 min [Fig. 1B,C; phosphorylated residue Ser477 (pS477) and phosphorylated residue Ser188 (pS188)], followed by full activation of cyclin-B–Cdk1, as indicated by complete dephosphorylation of Cdk1 [Fig. 1B,C; phosphorylation of residue Y15 (pY15)], resulting in GVBD at approximately 18 min. Subthreshold 1-MeAde induced Akt and Cdc25 phosphorylation within 2 min (Fig. 1B,C; pS477, pS188). At 8 min, we observed a decrease in Cdk1 phosphorylation on Tyr15, indicating initiation of cyclin-B–Cdk1 activation. Simultaneously, however, residue Ser188 of Cdc25 was rapidly dephosphorylated to basal levels, suggesting that signaling had been disrupted. Consistent with this, Cdk1 phosphorylation on Tyr15 returned to a level similar to that in immature oocytes, indicating that cyclin-B–Cdk1 was inactivated without reaching maximum activity. This partial and transient activation of cyclin-B–Cdk1 was confirmed by performing a direct kinase assay using histone H1 as a substrate (Fig. 1D,E). At 14 min, Cdc25 was again weakly phosphorylated and then gradually dephosphorylated to basal levels (Fig. 1B,C; Fig. S2A,B; pS188).

Next, in order to examine whether a characteristic dephosphorylation after subthreshold 1-MeAde stimulus is specific to Ser188 of Cdc25, we used glutathione S-transferase (GST)-conjugated peptide substrates of Akt, which are the Myt1-S75-peptide and another Akt substrate peptide (AS-peptide) derived from Cdc25 (residues Ala181–Gly193) in which the Akt consensus region was replaced with another efficient Akt substrate sequence RPRAATF (Alessi et al., 1996). Immature oocytes were injected with these peptides, then treated with a subthreshold concentration of 1-MeAde. As a result, a characteristic phosphorylation and subsequent dephosphorylation of the peptides were observed, as well as of Ser188 of Cdc25 (Fig. S2C–F). We also used a pan antibody that recognizes phospho-Akt substrates (PASs) with phosphorylated Ser and Thr residues in the Akt consensus site. Following subthreshold stimulus, this antibody detected multiple bands at 2 min, many (but not all) of which grew weaker or were lost in a manner concomitant with the characteristic Cdc25 dephosphorylation (Fig. 1B; PAS). Thus, we concluded that the characteristic dephosphorylation occurs on a wide range of Akt substrates, including Cdc25 and Myt1. Taken together, these findings indicate that although subthreshold stimuli can cause Akt activation and subsequent partial cyclin-B–Cdk1 activation, rapid Akt substrate dephosphorylation disrupts signaling.

Remarkably, the dynamics of Akt Ser477 phosphorylation were distinct from those of Cdc25 phosphorylation, peaking at 4 min and then decreasing gradually to near-basal levels (Fig. 1B,C; Fig. S2A,B; pS477). In addition, when we monitored phosphorylation of the PDK1-target site on Akt using exogenously introduced human Akt1 as we have reported previously (Hiraoka et al., 2011), we found that the dynamics of phosphorylation were similar to those of the phosphorylation of the TORC2-target site [Fig. S2G,H; phosphorylation of residue Thr308 (pT308), PDK1-target site; phosphorylation of residue Ser473 (pS473), TORC2-target site]. Thus, the characteristic rapid dephosphorylation of Akt substrates seems to occur while Akt remained active (at least at a level that can induce Cdc25 phosphorylation, Fig. 1B,C; compare pS477 at 2 min with 10 min).

A possible mechanism for the characteristic dephosphorylation of Akt substrates could be activation of phosphatases. We examined a phosphatase activity for Akt substrates in starfish oocytes. When the Myt1-S75-peptide or AS-peptide was phosphorylated by human Akt1 *in vitro* and then injected into immature oocytes, these peptides were dephosphorylated, indicating existence of a phosphatase activity even in unstimulated oocytes (Fig. 1F,G; ii, Myt1-S75-peptide; Fig. S2I for AS-peptide). Then we compared the rate of this dephosphorylation with that observed after subthreshold 1-MeAde stimulus (in this situation, unphosphorylated peptide was once phosphorylated in the oocytes and then subjected to dephosphorylation) by performing sampling every 1 min (Fig. 1F,G; i). The rate of dephosphorylation was faster after subthreshold 1-MeAde stimulus than in unstimulated oocytes (Fig. 1G; i versus ii, black lines), suggesting that a phosphatase was activated during the characteristic dephosphorylation event.

### Dephosphorylation of Akt substrates after subthreshold stimuli depends on partially activated cyclin-B–Cdk1

**Fig. 2.**
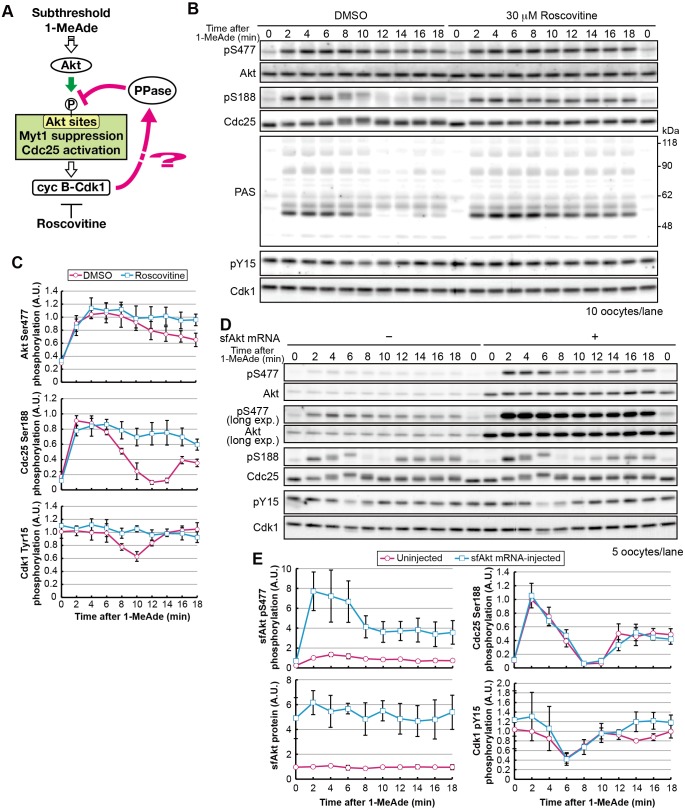
**The characteristic dephosphorylation of Akt substrates after treatment with subthreshold concentrations of 1-MeAde depends on cyclin-B–Cdk1 activity.** (A) Hypothesis tested in B and C. (B) Immature oocytes were treated with subthreshold levels of 1-MeAde (30 nM) in the presence of 30 μM roscovitine or 0.15% DMSO (as a negative control) and collected at the indicated times, followed by immunoblotting to detect phosphorylation and total protein levels. (C) Phosphorylation levels were quantified from the images in B, as described in Fig. 1C. Data represent mean values±s.d. from three independent experiments. (D) Immature oocytes were injected with mRNA encoding starfish Akt (sfAkt), incubated for 3 h, treated with a subthreshold concentration (30 nM) of 1-MeAde, and collected at the indicated times, followed by immunoblotting for phosphorylation and total proteins. (E) Phosphorylation levels were quantified from the images in D and normalized against the total amounts of each protein. Phosphorylation levels relative to those in uninjected oocytes at 2 min are shown. Data represent mean values±s.d. from three independent experiments. cyc, cyclin; exp., exposure; PPase, phosphatase.

Because the peak of partial cyclin-B–Cdk1 activation preceded Cdc25 dephosphorylation, we hypothesized that cyclin-B–Cdk1-dependent negative feedback was occurring. To test this idea, we used the Cdk-inhibitor roscovitine (Fig. 2A). After subthreshold 1-MeAde treatment in the presence of roscovitine, phosphorylation of Ser188 on Cdc25 and Akt substrates, detected by using the antibody against PASs, was maintained (Fig. 2B,C; pS188 and PAS). A similar effect was observed when yeast Suc1 protein was used to inhibit cyclin-B–Cdk1 (Fig. S3A). These results suggest that cyclin-B–Cdk1 causes Akt substrate dephosphorylation in order to prevent signaling after subthreshold stimuli. Because Ser477 phosphorylation on Akt was slightly elevated through inhibition of cyclin-B–Cdk1 (Fig. 2B,C; Fig. S3A), we examined whether this increase in Ser477 phosphorylation was sufficient to maintain Akt substrate phosphorylation. Following injection of starfish Akt mRNA, oocytes were treated with a subthreshold dose of 1-MeAde (Fig. 2D,E). The total protein and phosphorylation levels of Akt were increased approximately fivefold. However, the characteristic dephosphorylation of Akt substrates occurred as efficiently as in uninjected oocytes (Fig. 2D,E, pS188; see also Fig. S3B for PAS), suggesting that loss of the characteristic dephosphorylation event by roscovitine could not be attributed solely to increased phosphorylation of Ser477 on Akt.

### PI3K–Akt activity alone is not sufficient for phosphorylation of Akt substrates and meiotic G2/M transition

**Fig. 3.**
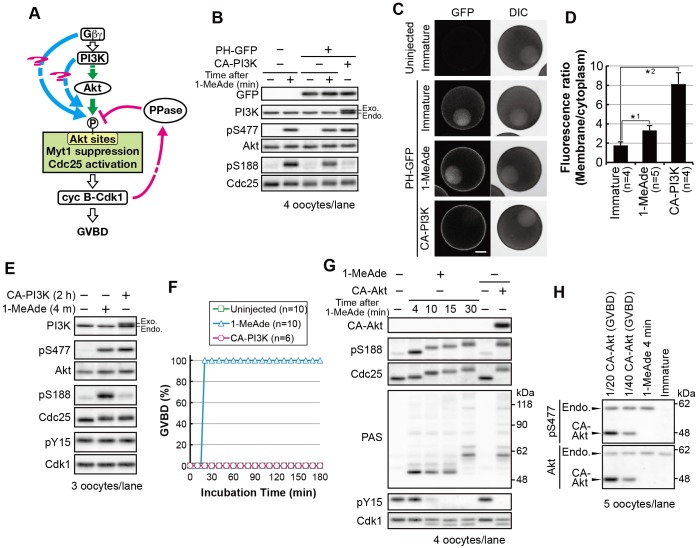
**PI3K–Akt activation alone is not sufficient to cause phosphorylation of Ser188 on Cdc25 and meiotic G2/M transition.** (A) A working hypothesis of the overriding pathways (cyan). (B–D) Immature oocytes were injected with mRNA encoding PH–GFP. After 1.5 h, a group of these oocytes were further injected with mRNA encoding FLAG-tagged CA-PI3K, then incubated for 5 h. Another group of oocytes injected with mRNA encoding PH–GFP were incubated for 6.5 h and then treated with a supra-threshold concentration of 1-MeAde (500 nM) for 4 min. These oocytes were analyzed by immunoblotting (B). Fluorescence images of oocytes treated under the same conditions as in B were obtained with confocal laser microscopy analysis (C). Typical fluorescence images of the oocytes are shown. The focal plane was set on the equatorial plane. Note that in all cases, oocytes had intact germinal vesicles in which some PH–GFP had accumulated. Scale bar: 50 μm. The plasma-membrane-to-cytoplasm fluorescence density ratio was calculated for each oocyte from the fluorescence images, as shown in C. Mean values of the ratio are indicated in D. *n* indicates the number of oocytes observed. Error bars represent the s.d. *P*-values (one-tailed *t*-test): *1, *P*<10^−3^; *2, *P*<10^−4^. (E,F) After the addition of a supra-threshold concentration of 1-MeAde or injection of mRNA encoding FLAG–CA-PI3K, immunoblotting was performed (E) or the GVBD ratio was determined (F). *n* indicates the number of oocytes observed. (G) Immature oocytes were injected with mRNA encoding CA-Akt and then collected immediately after GVBD (approximately 3 h after injection), or were treated with a supra-threshold (500 nM) concentration of 1-MeAde, followed by immunoblotting. (H) To compare expression levels of CA-Akt with those of endogenous Akt, the sample of CA-Akt-expressing oocytes immediately after GVBD were diluted 20- or 40-fold (1/20 or 1/40) with the sample of 1-MeAde-treated (500 nM, 4 min) oocytes and were then analyzed by immunoblotting. Endo, endogenous; exo, exogenous.

Cyclin-B–Cdk1-dependent negative feedback should be present even when oocytes receive supra-threshold doses of 1-MeAde. Thus, to achieve meiotic G2/M-phase transition in response to supra-threshold stimuli, oocytes must override this pathway. The 1-MeAde dose-dependent increase in Akt phosphorylation on Ser477 (see Fig. 1C; pS477 increases less than twofold) is less likely to be sufficient to override the negative feedback mediated by cyclin-B–Cdk1 because even a fivefold increase in the amount of Akt as a result of exogenous Akt expression failed to prevent Cdc25 dephosphorylation (see Fig. 2D,E). Therefore, another mechanism could exist. Because microinjection of G_βγ_ protein induces GVBD in starfish oocytes (Chiba et al., 1993; Jaffe et al., 1993), we hypothesized that the pathway necessary to override the cyclin-B–Cdk1-dependent negative feedback is activated by G_βγ_ or its downstream factors, such as PI3K (Fig. 3A).

First, we tested whether activation of PI3K is sufficient to induce meiotic G2/M transition. We constructed a constitutively active form of PI3K (CA-PI3K). We confirmed PIP_3_ production using a GFP fusion of the pleckstrin homology (PH) domain of starfish Akt (PH–GFP), which binds to PIP_3_ (Hirohashi et al., 2008). A greater increase in the ratio of GFP fluorescence in the plasma membrane to that in the cytoplasm was detected after CA-PI3K expression, through mRNA injection, than after 1-MeAde treatment (4.6-fold and 1.9-fold, respectively), indicating that more PIP_3_ was produced at the plasma membrane through CA-PI3K expression (Fig. 3B–D). Immunoblot analysis showed that CA-PI3K induced Akt phosphorylation on Ser477 at a level comparable to that induced by supra-threshold levels of 1-MeAde (Fig. 3E). In addition, when monitored on human Akt1, phosphorylation of the PDK1-target site was also induced (Fig. S4A, pT308). Nevertheless, no or less phosphorylation of Akt substrates was observed in CA-PI3K-expressing oocytes (Fig. 3E; pS188). Furthermore, CA-PI3K failed to induce GVBD (Fig. 3F). In contrast, we have previously reported that constitutively active Akt (CA-Akt) induces GVBD (Hiraoka et al., 2011; Okumura et al., 2002). However, based on our re-evaluation, extremely high activity (40-fold higher than that of endogenous Akt) is required for induction of Akt substrate phosphorylation and GVBD by CA-Akt alone (Fig. 3G,H). These results suggest that activation of the PI3K–Akt pathway alone is not sufficient for full phosphorylation of Akt substrates and meiotic G2/M transition.

### G_βγ_ activates an atypical pathway that enhances PI3K–Akt-dependent Akt substrate phosphorylation to induce meiotic G2/M transition

**Fig. 4.**
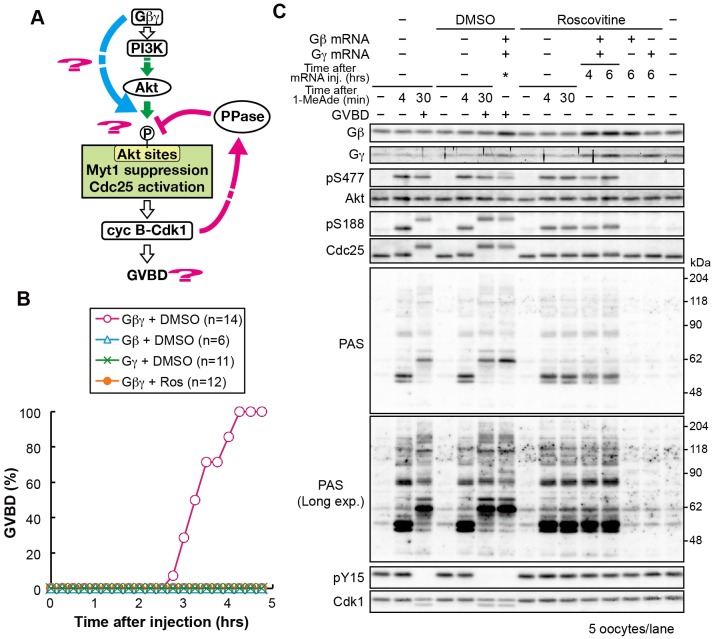
**Exogenous G_βγ_ expression mimics 1-MeAde-stimulated signaling.** (A) Hypothesis tested in B and C. (B) Immature oocytes were injected with mRNA encoding either untagged G_β_ or G_γ_, or an equimolar mixture of both mRNAs followed by incubation with 30 μM roscovitine (Ros) or 0.15% DMSO as a control. The proportion of oocytes that had undergone GVBD was determined following incubation for the indicated times. *n* indicates the number of oocytes observed. (C) G_β_, G_γ_ or G_βγ_ was expressed with roscovitine, as described in B. The oocytes were collected at the indicated times after mRNA injection. G_βγ_-expressing oocytes that had been incubated with DMSO were collected immediately after GVBD (asterisk; approximately 3–4 h). For 1-MeAde-treated oocyte samples, immature starfish oocytes were incubated with roscovitine or DMSO for 6 h and then treated with a supra-threshold (500 nM) concentration of 1-MeAde for the indicated times. In the absence of roscovitine, GVBD occurred approximately 22 min after 1-MeAde addition. Samples were analyzed by immunoblotting to detect phosphorylation and total protein.

Next, we investigated signaling from G_βγ_ (Fig. 4A). Expression of exogenous G_βγ_ complex, but neither the G_β_ nor G_γ_ subunits alone, induced GVBD (Fig. 4B). The amount of exogenously expressed G_βγ_ was physiologically relevant – mRNA injection of the starfish G_β_- and G_γ_-encoding genes resulted in an approximately twofold increase in G_βγ_ protein, implying that exogenous G_βγ_ was expressed at a level similar to that of the endogenous proteins (Fig. 4C). Immunoblot analysis of the oocytes immediately after GVBD showed that phosphorylation of Akt and its substrates was similar to that induced by supra-threshold levels of 1-MeAde (Fig. 4C; pS477, pS188 and PAS). Such similarity between 1-MeAde stimulation and G_βγ_ expression was also observed when cell cycle progression was inhibited with roscovitine (Fig. 4B,C). Furthermore, the PI3K inhibitor wortmannin abolished all phosphorylation events (Fig. S4B). These observations suggest that signaling from G_βγ_ is sufficient to mimic 1-MeAde signaling, leading to full activation of cyclin-B–Cdk1 that is dependent on the PI3K–Akt pathway. Considering that CA-PI3K expression was not sufficient for phosphorylation of Akt substrates or meiotic G2/M transition, it is likely that G_βγ_ activates not only the PI3K–Akt pathway but also another pathway (here referred to as an ‘atypical G_βγ_ pathway’) that enhances the PI3K–Akt-dependent phosphorylation of Akt substrates to induce the meiotic G2/M transition.

### Expression of mutant G_βγ_ lowers the 1-MeAde threshold

**Fig. 5.**
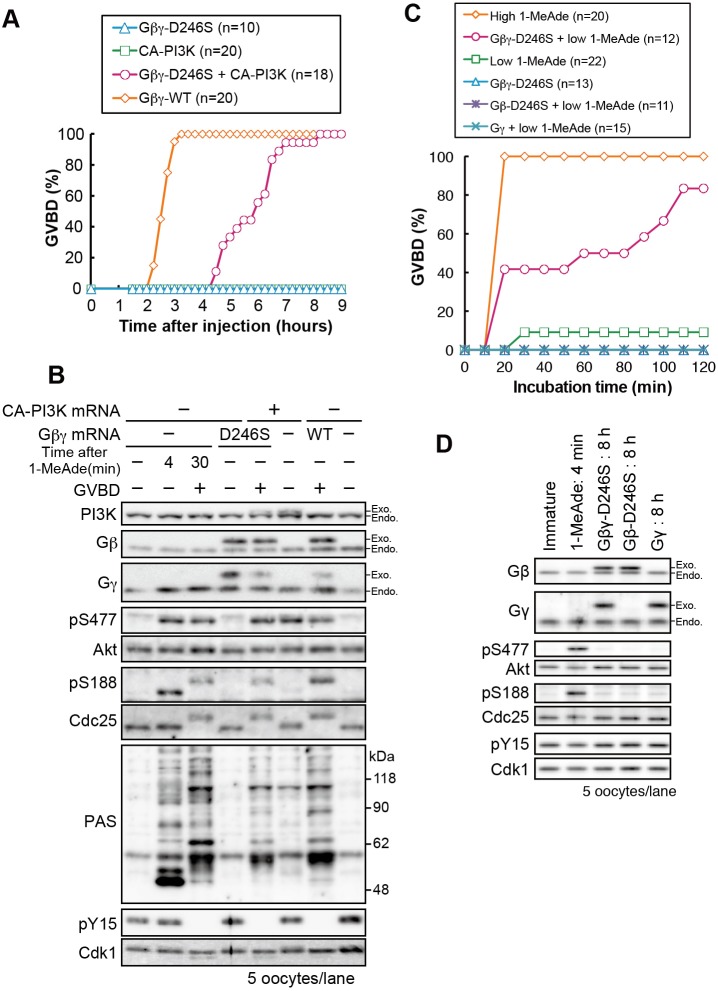
**G_βγ_ activates a pathway that is distinct from, but works cooperatively with, the PI3K–Akt pathway to induce the meiotic G2/M-phase transition by enhancing Akt substrate phosphorylation.** (A) Equimolar mixtures of mRNAs encoding starfish FLAG–G_γ_ and either Myc-tagged wild-type G_β_ (G_βγ_-WT) or the D246S mutant G_β_ (G_βγ_-D246S) were injected into immature oocytes. mRNA encoding CA-PI3K was injected or co-injected with the G_βγ_-D246S mRNA mixture. The proportion of oocytes that had undergone GVBD was determined following incubation for the indicated times. (B) The oocytes described in A were collected 7 h after injection or immediately after GVBD. For the 1-MeAde-treated samples, immature oocytes were treated with a supra-threshold concentration (500 nM) of 1-MeAde for the indicated times. GVBD occurred approximately 20 min after 1-MeAde addition. The oocytes were then analyzed by immunoblotting. (C) mRNA encoding either FLAG–G_γ_ or Myc–G_β_-D246S, or an mRNA mixture of them (G_βγ_-D246S) was injected into immature oocytes. After 8 h, a subthreshold concentration (3 nM; low) of 1-MeAde was added. Then, the number of oocytes displaying GVBD was counted at the indicated times. (D) mRNA-injected oocytes (8 h) in C and oocytes treated with a supra-threshold concentration of 1-MeAde (4 min) were analyzed by immunoblotting. *n* indicates the number of oocytes observed (A,D). Data are representative of two independent experiments.

To test whether the atypical G_βγ_-pathway can act as an overriding mechanism in establishment of the threshold for 1-MeAde to induce the meiotic G2/M transition, first we attempted to construct a G_βγ_ mutant that activates the atypical pathway without activating the PI3K–Akt pathway. To this end, we introduced point mutations into G_β_ at residues involved in interactions between G_βγ_ and its target proteins (Ford et al., 1998; Li et al., 1998), and we found that the D246S mutant produced the desired phenotype. Expression of FLAG-tagged G_β_-D246S with Myc-tagged G_γ_ (referred to as the mutant G_βγ_) failed to induce Akt phosphorylation, Akt substrate phosphorylation and GVBD (Fig. 5A,B). Nevertheless, co-expression of the mutant G_βγ_ with CA-PI3K resulted in Akt substrate phosphorylation and GVBD (Fig. 5A,B). Then, we treated the mutant-G_βγ_-expressing oocytes with subthreshold levels of 1-MeAde. As a result, these oocytes underwent GVBD, suggesting that an increase in activity of the atypical G_βγ_-pathway is likely to override cyclin-B–Cdk1-dependent negative feedback (Fig. 5C,D).

### The atypical G_βγ_ pathway is required for PI3K–Akt-dependent phosphorylation of Cdc25 and Myt1, even in the absence of cyclin-B–Cdk1-dependent negative feedback

Considering G_βγ_-dependent signaling is first activated before cyclin-B–Cdk1 activation, the atypical G_βγ_ pathway might not only act as overriding machinery against cyclin-B–Cdk1-dependent negative feedback but could also play a positive role in phosphorylation of Akt substrates, even in the absence of this negative-feedback pathway.

**Fig. 6.**
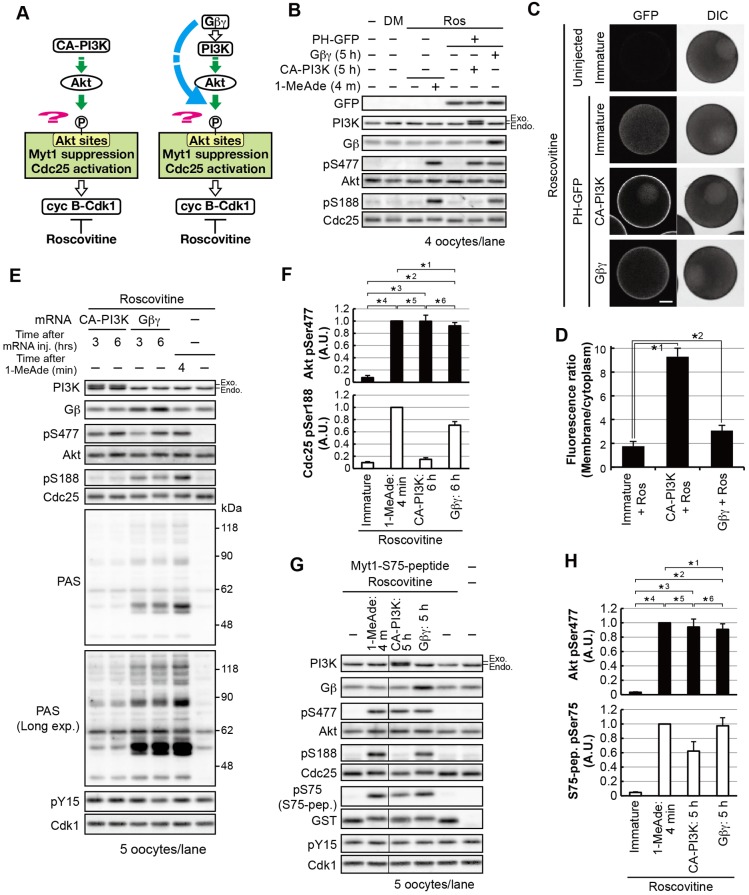
**The PI3K–Akt pathway is not sufficient for phosphorylation of Akt substrates, including Cdc25 and Myt1, but the atypical G_βγ_ pathway enhances phosphorylation even in the absence of cyclin-B–Cdk1-dependent negative feedback.** (A) Hypothesis tested in B–H. (B–D) PH–GFP was expressed in immature oocytes, followed by expression of either FLAG–CA-PI3K or G_βγ_ through incubation with roscovitine for 5 h after mRNA injection. For treatment with 1-MeAde, immature oocytes were pre-incubated with roscovitine, then treated with a supra-threshold concentration of 1-MeAde for 4 min. The oocytes were analyzed by immunoblotting (B) or confocal laser microscopy (C). In all cases, each oocyte had an intact germinal vesicle, within which some PH–GFP had accumulated, although in some cases it was out of the focal plane. Scale bar: 50 μm. The plasma-membrane-to-cytoplasm ratio of fluorescence density is shown in D. Data represent mean values±s.d. (five oocytes for immature and G_βγ_, six oocytes for CA-PI3K). *P*-values (one-tailed *t*-test): *1, *P*<10^−8^; *2, *P*<10^−3^. (E,F) After treatment with a supra-threshold concentration of 1-MeAde (500 nM), or FLAG–CA-PI3K or G_βγ_ expression with roscovitine, oocytes were analyzed by immunoblotting (E). Phosphorylation levels relative to those after treatment with 1-MeAde were quantified. Data represent mean values±s.d. from three independent experiments (F). *P*-values (one-tailed *t*-test) for Akt: *1, *5, *6, *P*>0.05 (not significant, n.s.); *4, *P*<10^−3^; *2, *3, *P*<10^−4^. For Cdc25, *3, *P*<0.05; *1, *P*<0.01; *5, *P*<10^−3^; *2, *4, *6, *P*<10^−4^. (G,H) Immature oocytes were injected with the GST-Myt1-S75-peptide, followed by expression of either CA-PI3K or G_βγ_ by mRNA-injection, then incubated with roscovitine for 5 h. For 1-MeAde treatment, oocytes were treated with a supra-threshold (500 nM) concentration of 1-MeAde for 4 min after pre-incubation with roscovitine for 5 h. The oocytes were analyzed by immunoblotting (G). Phosphorylation levels relative to those after treatment with 1-MeAde were quantified. Data represent mean values±s.d. from three independent experiments (H). In F and H, the numbers showing comparisons (*1 to *6) apply to both the upper and the lower panels. *P*-values (one-tailed *t*-test) for Akt: *1, *5, *6, *P*>0.05 (n.s.); *2, *3, *P*<0.01; *4, *P*<10^−5^. For the Myt1-S75-peptide (S75-pep): *1, *P*>0.05 (n.s.); *5, *6, *P*<0.05; *2, *3, *P*<0.01; *4, *P*<10^−4^.

To test this idea, CA-PI3K or G_βγ_ were expressed, through injection of mRNA, in immature oocytes in the presence of roscovitine, which prevents activation of cyclin-B–Cdk1-dependent negative feedback (Fig. 6A). When PIP_3_ production was monitored by using PH-GFP, both CA-PI3K and G_βγ_ increased the membrane-to-cytoplasm fluorescence ratio (5.4- and 1.8-fold, respectively; Fig. 6B–D), indicating PIP_3_ production at the plasma membrane at higher (or at least comparable) levels than that induced by supra-threshold amounts of 1-MeAde (see Fig. 3C,D). Furthermore, both constructs induced phosphorylation of Akt at a similar levels to that induced by 1-MeAde-treatment (Fig. 6E–H; pS477). Nonetheless, CA-PI3K induced significantly less Akt substrate phosphorylation than G_βγ_ (Fig. 6E,F for pS188 and PAS, Fig. 6G,H for pS75; see Fig. S4C for DMSO or kinase-dead control of CA-PI3K). Taken together, these results suggest that even in the absence of activation of cyclin-B–Cdk1-dependent negative feedback, activation of the PI3K–Akt pathway alone is not sufficient for efficient phosphorylation of Akt sites on Cdc25 and Myt1, and that the atypical G_βγ_ pathway enhances these phosphorylation events.

### The phosphatase activity towards Akt substrates is not altered by the atypical G_βγ_ pathway

In order to upregulate phosphorylation levels of Akt substrates, the atypical G_βγ_ pathway might reduce phosphatase activity towards Akt substrates. Thus, we examined whether the atypical G_βγ_ pathway alters the phosphatase activity.

**Fig. 7.**
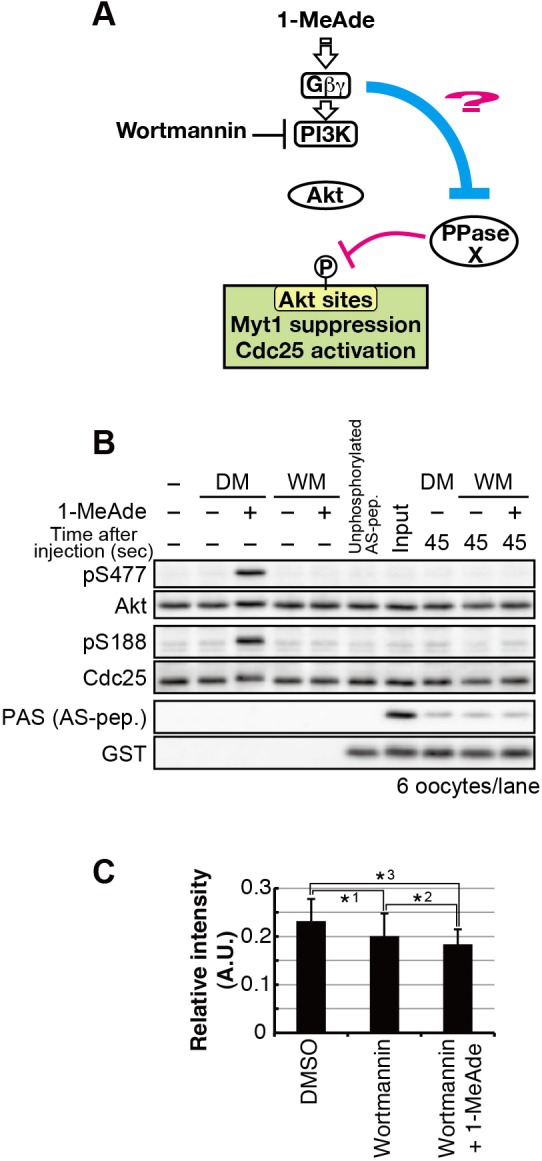
**The atypical** G**_βγ_ pathway does not alter phosphatase activity for Akt substrates in the absence of cyclin-B–Cdk1-dependent negative feedback.** (A) Hypothesis tested in B and C. (B,C) Immature oocytes after pre-incubation with 40 μM wortmannin (WM) or 0.2% DMSO (DM), or oocytes treated with a supra-threshold concentration of 1-MeAde (500 nM) for 4 min after pre-incubation with wortmannin were injected with the phosphorylated AS-peptide (AS-pep). Oocytes were collected at 45 s after injection, and analyzed by immunoblotting for phosphorylated and total protein (B). Phosphorylation of the AS-peptide (PAS) was quantified from images in shown in B and normalized against total protein (GST). Phosphorylation levels relative to those in the input are given as mean values±s.d. from three independent experiments (C). *P*-values (one-tailed *t*-test): *1, *2, *3, *P*>0.05 (n.s.).

To this end, the phosphatase activity for Akt substrates in immature oocytes was compared with that after 1-MeAde treatment in the presence of wortmannin (in this situation, it is assumed that 1-MeAde treatment activates the atypical G_βγ_ pathway while the PI3K-Akt-pathway is inhibited) (Fig. 7A). In order to evaluate the phosphatase activity in these oocytes, pre-phosphorylated AS-peptide was injected. Then dephosphorylation of the injected phospho-peptide in the oocytes was analyzed by immunoblotting. The AS-peptide was dephosphorylated to similar extents in both situations (Fig. 7B,C). This suggests that the atypical G_βγ_ pathway does not alter phosphatase activity towards Akt substrates, at least in the absence of cyclin-B–Cdk1-dependent negative feedback, and that the atypical G_βγ_ pathway can enhance Akt-catalyzed phosphorylation without affecting phosphatase activity.

## DISCUSSION

In this study, we identified two new pathways that regulate PI3K–Akt-pathway-dependent phosphorylation of Akt substrates in an opposing manner – the cyclin-B–Cdk1-dependent negative-feedback pathway, which induces dephosphorylation of Akt substrates and thereby prevents subthreshold stimulus-induced noise signaling, and an atypical G_βγ_-pathway, which is distinct from the PI3K–Akt pathway and enhances Akt-catalyzed phosphorylation. We also found that full activation of the PI3K–Akt pathway alone was not sufficient for efficient phosphorylation of Akt sites on Cdc25 and Myt1 regardless of activation of cyclin-B–Cdk1-dependent negative feedback. Cooperation of the atypical G_βγ_ pathway with the PI3K–Akt pathway first initiates activation of cyclin-B–Cdk1 and then overrides the negative feedback that arises as a result; thereby, inducing full activation of cyclin-B–Cdk1 and the meiotic G2/M transition.

In the context of threshold establishment, it is plausible to assume that the activity of the atypical G_βγ_-pathway depends on the dose of 1-MeAde – higher concentrations of the hormone induce dissociation of a larger number of G_βγ_ subunits, which in turn enhances activation of the atypical G_βγ_ pathway. The point at which activity of the atypical G_βγ_ pathway becomes predominant to that of cyclin-B–Cdk1-dependent negative feedback could be the threshold. Although we cannot yet directly test contribution of the atypical G_βγ_ pathway to threshold establishment with loss of function experiments, so far, our result that expression of the mutant G_βγ_-D246S lowers the threshold for 1-MeAde to induce the meiotic G2/M transition supports this idea.

### A model for threshold-setting for the meiotic G2/M transition

**Fig. 8.**
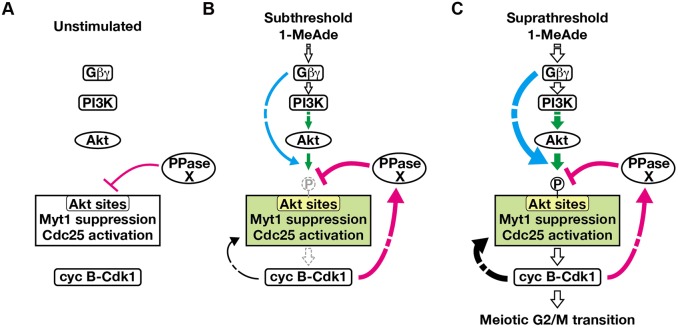
**A model for threshold-setting by noise canceling and overriding pathways in 1-MeAde signaling.** (A) In unstimulated immature oocytes, an unknown phosphatase for Akt substrates is active (PPase X), and all pathways shown in B and C with colors are inactive. (B) When oocytes receive a subthreshold 1-MeAde stimulus, the atypical G_βγ_ pathway (cyan) works cooperatively with the PI3K–Akt pathway (green) to phosphorylate Myt1 and Cdc25, thereby initiating activation of cyclin-B–Cdk1. However, cyclin-B–Cdk1 activates cyclin-B–Cdk1-dependent negative feedback (magenta), resulting in activation of an unknown phosphatase (note that here we drew this phosphatase as the PPase X, although it remains unclear whether they are the same molecule or not), thereby dephosphorylating Cdc25 and Myt1. Finally, cyclin-B–Cdk1 is inactivated by suppressive phosphorylation without reaching maximum activity. Although the cyclin-B–Cdk1-dependent positive-feedback pathway (black arrow from cyclin-B–Cdk1) might be partially activated, it is inactivated along with cyclin-B–Cdk1 before formation of an autonomously sustainable positive-feedback loop. (C) When oocytes receive supra-threshold 1-MeAde stimulus, the atypical G_βγ_ pathway and PI3K–Akt pathway activate cyclin-B–Cdk1. Although cyclin-B–Cdk1-dependent negative feedback might be active under these conditions, the atypical G_βγ_ pathway would be strongly activated enough to override this negative-feedback mechanism to maintain phosphorylation of Akt substrates. Thus, cyclin-B–Cdk1 reaches maximum activity through a fully functional positive-feedback loop, and oocytes irreversibly undergo the meiotic G2/M transition.

Based on present findings, we propose a model, shown in Fig. 8, for threshold-setting for the meiotic G2/M transition. In multiple scenarios that can be applicable to our results, we provide a simple one by introducing some speculation – first, the phosphatase activity towards Akt substrates detected in immature oocytes and in cyclin-B–Cdk1-dependent negative feedback events are attributed to the same molecule (an unknown phosphatase, referred to as PPase X); second, the atypical G_βγ_ pathway only enhances phosphorylation by Akt; third, the negative feedback resulting from cyclin-B–Cdk1 activation only activates PPase X. In unstimulated immature oocytes (Fig. 8A), all pathways are inactive, but an unknown phosphatase for Akt substrates, including Myt1 and Cdc25 (PPase X in Fig. 8), is active. Subthreshold stimuli (Fig. 8B) activate the PI3K–Akt pathway (Fig. 8; green) and the atypical G_βγ_ pathway (Fig. 8; cyan). Under these conditions, the atypical G_βγ_ pathway is activated to a level sufficient to override the PPase X basal activity but not to override the activity of PPase X that is enhanced by cyclin-B–Cdk1-dependent negative feedback. Thus, subthreshold signaling can initiate cyclin-B–Cdk1 activation, but this is reversed through the negative-feedback effects. The cyclin-B–Cdk1-dependent positive-feedback pathway (Fig. 8; black arrow from cyclin-B–Cdk1) (Kishimoto, 2015; Hara et al., 2012; Okumura et al., 2014) might be inactivated as a result of inactivation of cyclin-B–Cdk1, and thereby fail to form a strong positive-feedback loop. The activity of the atypical G_βγ_ pathway appears to depend on the dose of 1-MeAde. Supra-threshold doses of 1-MeAde sufficiently activate the atypical G_βγ_ pathway to override the cyclin-B–Cdk1-dependent negative feedback, thus maintaining phosphorylation of Akt sites on Cdc25 and Myt1 (Fig. 8C). Cyclin-B–Cdk1 then becomes maximally activated through a fully functional positive-feedback loop.

### Phosphatase for Akt substrates in starfish oocytes

We revealed an important role of phosphatase(s) in the regulation of phosphorylation levels of Akt substrates in starfish oocytes, although the enzyme responsible remains unclear. Protein phosphatase 1 (PP1) and protein phosphatase 2A (PP2A) are reported to dephosphorylate Akt sites on Bad and GSK3β, respectively (Ayllón et al., 2000; Sutherland et al., 1993). In addition, microinjection of okadaic acid, which inhibits PP1, PP2A, protein phosphatase 4 and protein phosphatase 6, induces the meiotic G2/M-phase transition in starfish oocytes (Picard et al., 1989). Thus, PP1 and PP2A could be the presumptive candidates for the Akt substrate phosphatases in starfish oocytes. Cyclin-B–Cdk1-dependent negative feedback seems to activate a phosphatase because the rate of dephosphorylation of the Myt1-S75-peptide was faster in the cyclin-B–Cdk1 negative-feedback pathway than that in immature oocytes. Generally, cyclin-B–Cdk1 is known to indirectly downregulate PP2A that contains the B55 regulatory subunit (PP2A-B55) through the inhibitory proteins Ensa or Arpp19 in M-phase (Castilho et al., 2009; Gharbi-Ayachi et al., 2010; Mochida et al., 2009, 2010; Vigneron et al., 2009). Thus, if PP2A is responsible for Akt substrate dephosphorylation in starfish oocytes, its regulatory mechanism would be distinct from that of the PP2A-B55 and Ensa (or Arpp19) system, rather using other regulatory subunits and regulatory proteins.

### Possible mechanisms of the cyclin-B–Cdk1-dependent negative feedback

The finding of negative feedback through cyclin-B–Cdk1 is surprising because all known cyclin-B–Cdk1-dependent feedback pathways are positive (Kishimoto, 2015; Lindqvist et al., 2009). The initial activation of cyclin-B–Cdk1 has been thought to lead to formation of a positive-feedback loop that accelerates activation of cyclin-B–Cdk1 itself. Our results show for the first time that cyclin-B–Cdk1 can put the brakes on its activation by itself.

In addition to upregulation of a phosphatase as discussed above, cyclin-B–Cdk1-dependent negative feedback might downregulate the kinase activity of Akt. As for Cdk-dependent regulation of Akt activity, Liu et al. have reported that cyclin-A–Cdk2 promotes Akt activation through direct phosphorylation of Akt proteins in mammalian cells (Liu et al., 2014). However, the phosphorylation sites are not conserved in starfish Akt, and the effect of the cyclin-B–Cdk1-dependent negative feedback on Akt is, if any, rather negative. Because phosphorylation of Akt by TORC2 is independent of phosphorylation by PDK1 (Hiraoka et al., 2011), occurrence of TORC2-mediated phosphorylation does not always ensure phosphorylation by PDK1. In addition, we can not directly evaluate the phosphorylation of the PDK1 site on endogenous Akt so far. Therefore, we cannot completely exclude the possibility that phosphorylation of Akt by PDK1 is negatively regulated by cyclin-B–Cdk1-dependent negative feedback. However, this mechanism is a less likely possibility because, in the present study, the dynamics of PDK1-mediated phosphorylation of exogenous human Akt1 were similar to those of TORC2-mediated phosphorylation in starfish oocytes.

Alternatively, Akt-binding proteins could be involved. For example, c-Jun NH_2_-terminal kinase (JNK)-interacting protein 1 (JIP1) binds to and inhibits Akt independently of Akt phosphorylation in human prostate adenocarcinoma cells (Song and Lee, 2005). This type of Akt regulator could function in cyclin-B–Cdk1-dependent negative feedback. Furthermore, another possibility that the cyclin-B–Cdk1-dependent negative feedback might downregulate the atypical G_βγ_ pathway should be considered.

### Possible mechanisms for the atypical G_βγ_ pathway

A remarkable observation in present study is that activation of PI3K–Akt alone is not enough to cause Akt substrate phosphorylation, even in the absence of activation of the cyclin-B–Cdk1-dependent negative-feedback pathway, suggesting that Akt requires the input of additional mechanisms in order to phosphorylate its substrates in intracellular environments. When the PI3K–Akt pathway is activated by G_βγ_ through G-protein-coupled receptors, such as endothelin or serotonin receptors (Liu et al., 2003; Liu and Fanburg, 2006), the atypical G_βγ_ pathway would be activated and required for Akt to execute its cellular function. The molecular identity of the supporting pathway might be different depending on cell and ligand type.

Although the molecular details of the atypical G_βγ_ pathway are unclear, our results indicate that this pathway can work independently of cyclin-B–Cdk1-dependent negative feedback and not alter the phosphatase activity towards Akt substrates. One possibility would be the upregulation of the kinase activity of Akt through Akt-binding regulators (upregulation of activators or downregulation of inhibitors).

So far, we cannot completely exclude another possibility that the atypical G_βγ_ pathway upregulates another kinase (not Akt) that could phosphorylate Akt substrates. Akt belongs to the AGC kinase family (Pearce et al., 2010). Some kinases in the family have redundant substrate specificity with Akt. Particularly, serum and glucocorticoid-regulated kinase (SGK) is reported to be activated by PDK1 and TORC2, depending on PI3K (García-Martínez and Alessi, 2008; Kobayashi and Cohen, 1999). Although a regulatory mechanism of SGK activity that fits with the atypical G_βγ_ pathway has not been reported so far, investigation of the involvement of this kinase could be interesting.

### Threshold-setting in *Xenopus* oocytes

*Xenopus* oocytes are a well-established model for studying meiotic G2/M transition (Haccard and Jessus, 2006). Cyclin-B–Cdk1 activity has been shown to be bistable due to positive feedback by using *Xenopus* egg extract (Novak and Tyson, 1993; Pomerening et al., 2003). In addition, mitogen-activated protein kinase (MAPK) activity, which is involved in meiotic resumption in *Xenopus* but not in starfish oocytes, has been reported to exhibit an all-or-nothing response to hormonal stimuli in *Xenopus* oocytes (Ferrell and Machleder, 1998). Furthermore, PKA-dependent phosphorylation of Arpp19 at residue Ser109 has been reported to prevent the meiotic G2/M transition. Dephosphorylation of this residue is required for the transition, and the extent of dephosphorylation is hormone-dose dependent (Dupré et al., 2014). These properties are important to ensure an appropriate response to maturation-inducing hormones. However, to our knowledge, signaling dynamics in response to subthreshold stimuli have never been investigated in the *Xenopus* system. Our study emphasizes the importance of examining these dynamics in the *Xenopus* model to more fully elucidate the network structure responsible for regulating the meiotic G2/M transition. However, the increased signaling complexity in *Xenopus* oocytes, which includes *de novo* synthesis of proteins – such as Mos – and multiple phosphorylation events, might present a challenge (Haccard and Jessus, 2006; Schmitt and Nebreda, 2002).

In summary, we demonstrate cyclin-B–Cdk1-dependent negative feedback, insufficiency of the PI3K–Akt pathway for inducing Akt substrate phosphorylation, and enhancement of Akt substrate phosphorylation through an atypical G_βγ_ pathway. These findings provide a model for establishing a hormonal dose threshold for cyclin-B–Cdk1 activation at the meiotic G2/M transition through regulating the phosphorylation of Akt sites on Cdc25 and Myt1. The features of these two new pathways refine our knowledge of the field of cell cycle control and signal transduction.

## MATERIALS AND METHODS

### Oocyte preparation

Starfish *Asterina pectinifera* (renamed *Patiria pectinifera* in the 2007 National Center for Biotechnology Information Taxonomy Browser) were collected during the breeding season and kept in laboratory aquaria that were supplied with circulating seawater at 14°C. Fully grown immature oocytes without follicle cells were released from isolated ovaries after treatment with Ca^2+^-free artificial seawater (Ookata et al., 1992). All treatments with 1-MeAde or other chemicals, as well as microinjections, were performed in artificial seawater at 23°C; 500 nM 1-MeAde was used as the supra-threshold stimulus. The subthreshold concentration of 1-MeAde (the highest concentration at which oocytes did not undergo GVBD) was determined for each animal and is indicated in the figure legends.

### Chemicals

Roscovitine (Calbiochem) and wortmannin (LC Laboratories; Woburn, MA) were dissolved in DMSO at 20 mM as a stock solution, and used at a final concentration of 30 μM and 40 μM, respectively.

### Buffers

Composition of buffers and reaction mixtures are shown in Table S1.

### cDNA cloning

cDNA clones of starfish G_β_ and G_γ_ were identified by performing homology searches in a cDNA library of starfish expressed sequence tags (ESTs) using human G_β1_ (encoded by *GNB1*) and human G_γ1_ (encoded by *GNGT1*), respectively, as queries, and verified by sequencing. PI3K is a heterodimer consisting of a catalytic subunit (p110) and an adaptor subunit (Vanhaesebroeck et al., 2010). In four reported isoforms of p110 (α, β, γ, δ, encoded by *PIK3CA*, *PIK3CB*, *PIK3CG* and *PIK3CD*, respectively), p110β and p110γ can be activated by G_βγ_ (Vanhaesebroeck et al., 2010). Because p110γ exists only in mammals, we isolated a cDNA encoding the starfish homolog of p110β from a total mRNA library of immature oocytes using the SMART RACE cDNA Amplification kit (Clontech). A partial cDNA fragment was isolated by performing reverse-transcriptase (RT)-PCR with degenerate primers. The 5′ and 3′ ends were identified by performing rapid amplification of cDNA ends (RACE) with specific primers. The full-length open reading frame was amplified by using RT-PCR and cloned into the pGEM-T Easy vector (Promega). All accession numbers and primer sequences are shown in Tables S2 and S3, respectively. All primers were synthesized by Eurofins Genomics (Tokyo, Japan).

### DNA constructs

DNA constructs used in this study are shown in Table S4.

### Protein preparation

To prepare the recombinant GST-fused Akt peptide substrates, *Escherichia coli* strain BL21-CodonPlus-RIL (Agilent Technologies) was transformed with the pGEX-4T-1 constructs. Expression of recombinant proteins was induced through a 2-h incubation with 1 mM isopropylthiogalactoside (IPTG), and proteins were purified using glutathione–Sepharose-4B (GE Healthcare). For *in vitro* phosphorylation, the peptides were dialyzed against Tris-buffered saline (TBS) using EasySep membrane (TOMY; Tokyo, Japan). Then, 240 μg of peptides were incubated in a 240-μl reaction mixture containing active human Akt1 for 3 h at 30°C. The reaction was stopped by addition of EDTA. The phosphorylated peptide was purified using glutathione–Sepharose-4B to remove human Akt1. For microinjection, sample concentration and buffer exchange (to PBS) were performed using a 10-k cutoff Vivaspin column (GE Healthcare). NP-40 was added at a final concentration of 0.1%. For His_6_–Cdc25, G_β_–His_6_ and p110β–His_6_*, E. coli* strain BL21 (DE3) (Invitrogen) was transformed with each pET21a construct. His_6_–Cdc25 and G_β_–His_6_ were purified from inclusion bodies under denaturing conditions (6 M urea) using Probond-Resin (Invitrogen). Purified His_6_–Cdc25 was refolded by performing stepwise reduction of the urea concentration with dialysis using EasySep for 2 h against TBS containing 4 M urea, for 2 h against TBS containing 2 M urea and then for 2 h against TBS without urea; the protein was then used for the *in vitro* phosphatase assay. Purified G_β_–His_6_ was dialyzed against PBS and used as the antigen. Starfish p110β–His_6_ was contained almost entirely in inclusion bodies. For antigen preparation, the protein was separated by SDS-PAGE, and the gels were stained with Coomassie brilliant blue R-250 (CBB). The band corresponding with p110β–His_6_ was excised from the gel and extracted using an Electro-Eluter instrument (Bio-Rad); it was then dialyzed against PBS. For GST-tagged starfish p110β, the *E. coli* BL21 strain was transformed with the pGEX-4T-1 construct. The expressed protein was almost entirely contained in inclusion bodies, thus separated by SDS-PAGE, transferred onto a PVDF membrane and stained with Ponceau S. The band representing GST–p110β was excised, destained with TBS and used for antibody purification. Recombinant fission yeast Suc1 protein was bacterially produced and purified as described previously (Kusubata et al., 1992), and then dissolved in 0.05% NP-40 at a concentration of 12.35 mg/ml for microinjection.

### Antibody generation

All antibodies in Table S5 were generated by immunizing rabbits, then purified using antigen. Antigens for immunization and antibody purification are indicated in Table S5. Control IgG for microinjection was purified from rabbit pre-immune serum using protein-A–Sepharose-4B (Sigma-Aldrich). For microinjection, concentration and buffer exchange (to PBS) were performed using a 50-k cutoff Vivaspin column. NP-40 was added at a final concentration of 0.1%.

### Microinjection

Microinjection was performed as described previously (Kishimoto, 1986). mRNAs for exogenous protein expression in starfish oocytes were transcribed from pSP64-S constructs using the mMESSAGE mMACHINE kit (Ambion), dissolved in water and injected into immature oocytes. Incubation time for protein expression (indicated in each legend) was determined on the basis of the time to induce GVBD or to induce comparable Akt phosphorylation to that by 1-MeAde. The amounts of injected mRNAs or proteins per oocyte are shown in Table S6.

### Immunoblotting

Oocytes were lysed by vortexing in Laemmli sample buffer, and then boiled for 5 min. The proteins were separated by performing SDS-PAGE (Laemmli, 1970) and transferred onto a PVDF membrane (Millipore) (Towbin et al., 1979). Membranes were blocked in a blocking buffer for detection of starfish phospho-Myt1 (at Ser75), starfish Myt1 and GST, but not for other proteins. The primary antibodies are indicated in the Table S7. Alkaline-phosphatase- or horseradish peroxidase (HRP)-conjugated secondary antibodies were used. Proteins reacting with the antibodies were detected as follows: for HRP, visualization with ECL prime (GE Healthcare), followed by acquisition of digital images on a LAS4000 imager (Fujifilm); for alkaline phosphatase, visualization with the BCIP/NBT phosphatase substrate system (Kirkegaard and Perry Laboratories, Gaithersburg, MD), followed by acquisition of digital images with an MP630 scanner (Canon; Tokyo, Japan). Quantification was performed using Multi Gauge software version 3.0 (Fujifilm). Graph drawing and linear approximation were performed using Microsoft Excel. Brightness and contrast were adjusted equally across whole images using Photoshop (Adobe).

### *In vitro* Cdc25 phosphatase assay

The phosphatase activity of Cdc25 was measured indirectly. For phosphorylation of Cdc25 by Akt, recombinant His_6_-tagged starfish Cdc25 was incubated with active human Akt1 in 6 μl of reaction mixture for 30 min at 30°C. Next, 1 μl of inactive cyclin-B–Cdk1 [100 ng; purified from immature starfish oocytes as described previously (Okumura et al., 1996)] was added. After additional incubation for 10 min at 30°C to allow activation of cyclin-B–Cdk1 by Cdc25, 4 μl of histone H1 mixture was added and incubated for an additional 20 min at 30°C to allow phosphorylation of histone H1 by activated cyclin-B–Cdk1. Finally, 11 μl of 2× Laemmli sample buffer was added, and the sample was boiled for 3 min. The samples were run on a 12% SDS-PAGE gel and stained with CBB. The gels were autoradiographed using an imaging plate on a BAS2000 scanner (Fujifilm).

### H1 kinase assay

7.5 μl of lysis buffer was added to 30 oocytes in 5 μl of artificial seawater. After one freeze–thaw cycle, oocytes were lysed by vortexing, followed by centrifugation (16,000 ***g***, for 15 min). 0.5 μl of supernatant was used for the H1 kinase assay. The residual supernatant was analyzed by immunoblotting. For the H1 kinase assay, the supernatant was mixed with 9.5 μl of reaction mixture containing histone H1 and incubated at 25°C for 15 min. The reaction was stopped by adding Laemmli sample buffer, followed by boiling. After SDS-PAGE analysis on a 12.5% acrylamide gel, phosphorylation of histone H1 was detected by autoradiography and quantified by performing liquid scintillation counting.

### Fluorescence imaging

Fluorescence images were obtained with the Fluoview FV1000 confocal microscope (Olympus) using a 10× objective lens (UPLSAPO 10×; Olympus). Living oocytes expressing PH–GFP in artificial seawater were set under the microscope. Fluoview software was used to acquire digital images from the photon detector of the FV1000 system. ImageJ software (National Institutes of Health) was used for quantification. Translocation of PH–GFP was quantitatively estimated as follows. First, a region of interest (ROI) was defined along the cell periphery (ROI-out). Next, another ROI was defined by reducing the size of the first ROI by 7 μm inward (ROI-in). We defined the area between ROI-out and ROI-in as the plasma membrane region and the area inside ROI-in as the cytoplasmic region. When the germinal vesicle was included in the focal plane, an ROI outlining the germinal vesicle was defined, and the area outside of this ROI and inside ROI-in was defined as the cytoplasmic region. Next, the fluorescence intensity and the area of each region were quantified. To avoid variation in total intensity in the cytoplasmic region resulting from the presence or absence of the germinal vesicle on the focal plane, we used the fluorescence intensity per area (fluorescence density) to calculate the ratio. In addition, the average autofluorescence density of intact immature oocytes, which do not express PH–GFP, was defined as the background and subtracted from the density of PH–GFP-expressing oocytes. Finally, the fluorescence density in the plasma membrane region was divided by that in the cytoplasmic region to calculate the plasma-membrane-to-cytoplasmic ratio. Translocation of PH–GFP was estimated by using the change in this ratio.

### Statistical analysis

Statistical significance was calculated using unpaired one-tailed Student's *t*-test in Microsoft Excel with StatPlus (AnalystSoft; Walnut, CA). Statistical significance was defined as *P*<0.05.
